# Preparation of Chitosan Composite Film Loaded with Chlorogenic Acid–Chitosan Oligosaccharide Nanoparticles and Its Application in Preservation of *Pleurotus geesteranus*

**DOI:** 10.3390/foods15020221

**Published:** 2026-01-08

**Authors:** Ning Xu, Liru Luo, Fang Wu, Dan Luo, Liguo Feng, Huan Lu

**Affiliations:** 1 Institute of Hunan Edible Fungi, Changsha 410013, China; xning89@163.com (N.X.); 13786113296@163.com (L.L.); wfang17@126.com (F.W.); 84403187@163.com (D.L.); feng13974982041@163.com (L.F.); 2 Institute of Edible Fungi, Shanghai Academy of Agricultural Sciences, Shanghai 201403, China

**Keywords:** nanoparticles, chlorogenic acid, composite film, *Pleurotus geesteranus*, preservation

## Abstract

To reduce the environmental impact of plastic packaging in the edible fungi supply chain, this study developed an edible natural chitosan composite film loaded with chlorogenic acid–chitosan oligosaccharide nanoparticles (CGA/COS NPs). The effects of CGA/COS NPs as additives on the structure and overall performances of chitosan-based films were systematically studied, and the application effect of nanoparticles/chitosan (NPs/CS) composite films in the preservation of *Pleurotus geesteranus* was explored. The results showed that the NPs had good compatibility with the film matrix, filled the voids of the chitosan matrix, enhanced the comprehensive performance of the film, and significantly improved the antioxidant activity of the film (DPPH free radical scavenging activity increased from 16.95% to 76.47%). Among all the films, the 5%NPs/CS composite film performed the best, not only having stronger barrier properties against moisture, oxygen, and ultraviolet rays, but also having the best thermal stability and mechanical properties, which can effectively extend the shelf life of *Pleurotus geesteranus*. This study developed a high-performance edible composite film, which provides a new path of great value for solving the preservation problem of perishable agricultural products such as *Pleurotus geesteranus* and promoting the innovative development of the green food packaging industry.

## 1. Introduction

*Pleurotus geesteranus* is an edible fungus (mushroom) characterized by a sweet, crisp texture and savory flavor. Rich in bioactive compounds, they exhibit antioxidant, antitumor, anti-inflammatory, hypoglycemic, hypolipidemic, and immune-enhancing properties [1], making them highly favored by consumers. Due to the strong respiratory metabolism and enzyme activity, *Pleurotus geesteranus* is prone to water loss, discoloration, deterioration, and other phenomena during post-harvest storage, which accelerates aging and loses commercial value. Currently, low-temperature refrigeration combined with film packaging is the most common preservation method for *Pleurotus geesteranus* on the market [2]. However, it was found in production that the respiration and transpiration of *Pleurotus geesteranus* easily formed water mist on the inner surface of the packaging film, resulting in condensation, which caused changes in the humidity inside the bag, thereby nourishing bacteria, leading to accelerated spoilage and shortening of shelf life [3]. At present, some synthetic plastic films made of polyethylene (PE) and other materials are often used for short-term storage and preservation during the transportation and sales of *Pleurotus geesteranus*. However, these synthetic plastic films have poor antibacterial effect and limited preservation effect, and at the same time, they will cause white pollution due to their difficulty in degradation [4]. Therefore, the preparation of new green packaging materials with excellent performance has become a hot spot in the preservation research of *Pleurotus geesteranus*.

Edible film is a film prepared and synthesized with natural edible biological macromolecular substances as the main matrix [5]. Chitosan (CS) is a natural polymer, non-toxic and harmless [6], with film-forming ability, and can be directly used as a film or coating without the need for a carrier matrix. A large number of studies have added different components such as plant essential oils, plant extracts, organic acids, and bacteriocins to the chitosan matrix to construct composite films for antiseptic and fresh-keeping treatment of fruits, vegetables, and meat products [7,8]. Chitosan oligosaccharide (COS) is a functional oligosaccharide obtained from the degradation of chitosan or chitin. It has a series of biological activities, such as antibacterial and antitumor cell growth [9], and has a wide range of potential application value in food, pharmaceutical, agricultural, and environmental fields [10].

Chlorogenic acid (CGA) is a phenolic acid compound condensed from caffeic acid and quinic acid, and has the characteristics of anti-oxidation, antibacterial, and antiviral [11]. Some scholars have used it for long-term preservation of melons, fruits, and vegetables, and achieved good results [12,13]. However, the molecular structure of chlorogenic acid includes three unstable parts: ester bond, unsaturated double bond, and multiple phenols. Therefore, the use of CGA is limited by its low bioavailability and stability [14,15].

Research indicates that nanoparticles (NPs) exhibit unique surface effects and quantum size effects. Encapsulating functional groups within nanoparticles or adsorbing them onto nanoparticle surfaces can enhance the stability of bioactive components and maximize their activity [16]. Moreover, nanoparticles have been demonstrated to improve the mechanical and barrier properties of films. Their small size and large specific surface area enhance the reaction between nanofillers and the film matrix [17]. They also enhance the physicochemical and functional properties of films due to their high matrix/component interface area and improved controlled release and stability of active agents [18].

In this paper, chitosan oligosaccharide and chlorogenic acid were used as the main materials, and chitosan oligosaccharide nanoparticles loaded with chlorogenic acid were prepared by the ionic cross-linking method. Chitosan was used as the matrix, and chitosan composite film loaded with chitosan oligosaccharide–chlorogenic acid nanoparticles was prepared by adding nanoparticles. The effects of different nanoparticle additions on the barrier, mechanical, and thermal stability of chitosan composite film were investigated. This study provides experimental data for the modification research and application of chitosan-based film by nanoparticles and also develops an edible composite film, which is expected to provide new ideas for the design of food packaging for *Pleurotus geesteranus* preservation.

## 2. Materials and Methods

### 2.1. Materials and Reagents

Chitosan (MW: 50,000–60,000, 95% degree of deacetylation), Sodium tripolyphosphate: Macklin Biochemical Co., Ltd., Shanghai, China; Chitosan oligosaccharide (MW: 1000, degree of polymerization: 2–8): Provided by Professor Liangbin Zeng of the Institute of Bast Fiber Crops, Chinese Academy of Agricultural Sciences; Chlorogenic acid: D&B Biological Science and Technology Co., Ltd., Shanghai, China; Glycerol (AR), Anhydrous ethanol (AR): Sinopharm Chemical Reagent Co., Ltd., Shanghai, China; DPPH: Solarbio Science & Technology Co., Ltd., Beijing, China.

### 2.2. Preparation of CGA/COS NPs

The preparation of NPs followed the experimental method reported by Nallamuthu et al. [19] with minor modifications. Weigh precisely 25 mg of chitosan oligosaccharides and dissolve it in 25 mL of ultrapure water. The pH of the solution was adjusted to 4.5–5.0 using 1% acetic acid solution (*v*/*v*) to prepare a chitosan oligosaccharide solution (1 mg/mL). The solution was stirred at 500 r/min using a magnetic stirrer. Subsequently, a specific amount of chlorogenic acid (1, 2, 3, and 4 mg, respectively) was added to the chitosan oligosaccharides solution and stirred for 30 min. Prepare a 1 mg/mL sodium tripolyphosphate (TPP) solution in ultrapure water, and add it dropwise to the mixture at a rate of 1 drop/2 s to achieve a chitosan oligosaccharide to TPP mass ratio of 9:2. Continue stirring at the same rate for 30 min to obtain a suspension of chitosan oligosaccharide nanoparticles loaded with chlorogenic acid. A blank nanoparticle was prepared using the same method without adding chlorogenic acid.

### 2.3. Preparation of NPs/CS Composite Film

The composite film preparation followed the method of Genovese et al. [20] with minor modifications. Weigh 2.0 g of chitosan and dissolve it in a 1% acetic acid solution (*v*/*v*). Stir the solution in a 65 °C water bath for 30 min to obtain a 2% chitosan solution (*w*/*v*). Add 30 wt% glycerol (in relation to chitosan mass) as a plasticizer to the chitosan solution, and stir it at 750 r/min for 15 min. During this period, add CGA/COS NPs suspensions at volume fractions of 1%, 3%, 5%, and 10% (Calculated based on the total volume of the final composite membrane solution) to the chitosan solution. After ultrasonic degassing for 30 min, pour 60 g homogenized solution into molds (Polystyrene Petri dish, diameter 13 cm). After drying at 30 °C for 48 h, remove the film and place it in a desiccator for 48 h to allow the film’s moisture content to reach a stable state, thereby ensuring the stability of the test data, and set aside for later use. Based on the volume fraction of the added NPs suspension, the composite films were designated as 1%NPs/CS, 3%NPs/CS, 5%NPs/CS, and 10%NPs/CS films. The chitosan film without added NPs suspension served as the blank control, denoted as CS.

### 2.4. Characterization

#### 2.4.1. Determination of Nanoparticle Size and Zeta Potential

Dilute the NPs suspension prepared in Section 2.2 and sonicate for 15 min prior to testing. The particle size, polydispersity index (PDI), and zeta potential of NPs were determined using a nanoparticle size and zeta potential analyzer (Bettersize BeNano 90 Zeta, Dandong, China), and the stability of NPs in the dispersant was analyzed.

#### 2.4.2. Transmission Electron Microscopy (TEM)

The morphology and size of the NPs samples were characterized by transmission electron microscopy (JEOL JEM-F200, Tokyo, Japan). Dilute the COS NPs and CGA/COS NPs suspensions 10-fold with ultrapure water. Deposit 10 μL of the diluted sample onto a copper grid and stain with 2% (*m*/*v*) phosphotungstic acid for 1 min. After drying at room temperature, observe the sample using TEM at an acceleration voltage of 120 kV. Based on the transmission electron microscopy imaging results, the NPs diameters were analyzed using Nano Measurer 1.2.5 software (Fudan University, Shanghai, China).

#### 2.4.3. Morphology of Composite Film

The morphology of the film’s front surface and cross-section was observed using scanning electron microscopy (SEM, ZEISS Sigma 300, Jena, Germany). The film was mounted on double-sided conductive adhesive film, and SEM images of the front surface were acquired at 3 kV acceleration voltage. The films were frozen in liquid nitrogen and then quickly impact fractured for cross-sectional observations. All samples were coated with a thin layer of gold to enhance surface conductivity for SEM observation.

#### 2.4.4. Fourier Transform Infrared Spectroscopy

Functional groups within the samples were identified using a Fourier Transform Infrared (FTIR) spectrometer (Thermo Fisher Scientific Nicolet iS20, Waltham, MA, USA) in transmission mode. Fourier Transform Infrared spectra were recorded in the 4000–400 cm^−1^ range using Attenuated Total Reflection (ATR) mode to investigate the interaction between the CS film matrix and the nanoparticles (NPs). The type of crystal is diamond, the number of scans is 16, and the resolution ratio of measurements is 3.818.

#### 2.4.5. X-Ray Diffraction

XRD analysis was performed using an X-ray diffractometer (Rigaku SmartLab SE, Takatsuki, Osaka, Japan) at 40 kV, 20 mA. XRD patterns were collected at a rate of 2°/min over the 2θ range of 5–40°.

#### 2.4.6. Thermogravimetric Analysis (TGA)

Thermogravimetric curves of the films were measured using a thermogravimetric analyzer (TA Q500, Newcastle, DE, USA). Use the films that were placed in the desiccator for 48 h of equilibration in Section 2.3. Approximately 20 mg of film was weighed and heated to 600 °C at a heating rate of 10 °C/min under a nitrogen atmosphere, determining the thermal stability of films.

#### 2.4.7. Static Contact Angle

The static contact angle of the film at room temperature was measured using a contact angle meter (Shengding SDC 350KS, Kunshan, China). Use the films that were placed in the desiccator for 48 h of equilibration in Section 2.3. A drop (2 μL) of deionized water was dropped on the surface of the thin film, and the image of the water drop was recorded and the contact angle value was calculated at the moment when the water drop contacted the surface of the thin film.

#### 2.4.8. Mechanical Properties

Tensile tests were performed using a universal testing machine (Hongtuo Instrument HT-101SC-5, Dongguan, China) equipped with a 500 kg load cell. Use the films that were placed in the desiccator for 48 h of equilibration in Section 2.3. Prior to testing, film samples were cut into rectangular strips (6 cm × 1 cm) and clamped in the fixture with an initial gauge distance of 30 mm. Analysis was conducted at a test speed of 0.5 mm/s until film rupture.

#### 2.4.9. Water Content, Swelling Degree, and Water Solubility

Water content (WC), swelling degree (SD), and water solubility (WS) were determined according to the method of Yao, Wang, and Weng et al. [21]. Use the films that were placed in the desiccator for 48 h of equilibration in Section 2.3. Film samples (2 cm × 2 cm) were cut and weighed to obtain M_0_. They were then dried in an oven (105 °C) for 24 h and weighed again to obtain M_1_. Subsequently, the dried film was immersed in 30 mL of ultrapure water for 24 h (25 °C), weighed, and recorded as M_2_. No residual moisture remained on the film surface. The film was then dried again at 105 °C for 24 h, weighed, and recorded as M_3_. WC, SD, and WS were calculated as follows (1), (2), and (3):

(1) $$\mathrm{WC}\;(\%)\;=\;\frac{M_{0}-M_{1}}{M_{0}}\times 100$$

(2) $$\mathrm{SD}\;(\%)=\frac{M_{2}-M_{1}}{M_{1}}\times 100$$

(3) $$\mathrm{WS}\;(\%)=\frac{M_{1}-M_{3}}{M_{1}}\times 100$$

#### 2.4.10. Oxygen Transmission Rate (OTR) and Water Vapor Permeability (WVP)

The OTR value of the film was measured using a permeability tester (Labthink BTY-B2P, Jinan, China) at room temperature (about 23 °C) and 50% relative humidity. The oxygen transmission rate was measured according to the national standard GB/T1038.1-2022 [22]. The water vapor permeability (WVP) of the film was measured strictly in accordance with the national standard GB/T 1037-2021 [23]. Prior to testing, place the moisture-permeable cup containing sealed desiccant (calcium chloride) within a constant temperature and humidity chamber maintained at 23 °C and 90% relative humidity for 48 h of pre-conditioning, allowing the specimen to reach moisture equilibrium with the environment. Following pre-conditioning, weigh the moisture-permeable cup at 24 h intervals. The formula for calculating the WVP is as follows:

(4) $$\mathrm{WVP}\;(g\text{·}\mathrm{mm}/(m^{2}\text{·}d\text{·}\mathrm{kPa}))\;=\;\frac{\Delta_{m}\times D}{A\times T\times \Delta_{p}}$$

∆_m_: Mass change in the moisture permeation cup during time interval t, g; D: Specimen thickness, mm; A: Effective moisture permeation area of the specimen, m^2^; T: Difference in weighing time intervals after mass change stabilization, taken as 24 h (i.e., 1 day); ∆_p_: Water vapor pressure difference across the specimen, KPa.

#### 2.4.11. Optical Properties

The optical transmittance of film samples was measured from 200 to 800 nm by using a UV−vis near-infrared (NIR) spectrophotometer (Shimadzu UV-3600i Plus, Kyoto, Japan). Additionally, the opacity values of these samples were measured at a wavelength of 600 nm, calculated as follows:

(5) $$\mathrm{Opacity}\;=\;\frac{A_{600}}{T_{d}}$$

A_600_: The film absorbance at 600 nm; T_d_: The film thickness, mm.

#### 2.4.12. Antioxidant Activity

DPPH radical scavenging assay was obtained by the method of Hosseini et al. [24]. 0.5 g of the film was weighed and immersed in 50 mL of 80 vol% ethanol, and centrifuged at 3000 r/min for 30 min at 25 °C. Then, 2 mL of the ethanol film solution was added to 2 mL of DPPH (0.2 mmol/L) and placed in the dark for 30 min. The absorbance was measured at 517 nm using a multi-mode microplate reader (perkinelmer victor nivo, Norwalk, CT, USA), and the DPPH scavenging activity was calculated according to Formula (6):

(6) $$\mathrm{DPPH}\;\mathrm{Scavenging}\;\mathrm{activity}\;(\%)\;=\;(1-\frac{A_{s}-A_{b}}{A_{0}})\times 100$$

A_s_: Absorbance of the supernatant after reaction with DPPH ethanol solution; A_b_: Absorbance of the supernatant after mixing with anhydrous ethanol; A_0_: Absorbance of the mixture of 80 vol% ethanol solution and DPPH ethanol solution.

### 2.5. Applications on Pleurotus geesteranus

#### 2.5.1. Freshness Preservation Test

Fresh *Pleurotus geesteranus* with uniform size, no disease, insect pests, and mechanical damage were selected and divided into 4 equal parts. They were set as the control group, ordinary PE packaging group, CS film group, and NPs/CS composite film group, respectively. They were placed in transparent plastic boxes (11.8 cm × 8.3 cm × 4.3 cm) without lids and sealed at the top with the prepared films, and stored in a refrigerator at (4 ± 1) °C (85% RH) for 10 d.

#### 2.5.2. Weight Loss Rate

The mass loss rate of *Pleurotus geesteranus* during storage was determined using the weighing method [25]. All of the samples from each group were weighed on different days, and the mass loss rate was calculated according to Equation (7):

(7) $$\mathrm{Weight}\;\mathrm{loss}\;(\%)\;=\;\frac{M_{0}-M_{n}}{M_{0}}\times 100$$

where M_0_ is the initial weight of the *Pleurotus geesteranus*, and M_n_ is the weight of the *Pleurotus geesteranus* on day n.

### 2.6. Statistical Analysis

In this experiment, data processing and graphing were performed using Origin 2018 software, while data analysis was conducted with SPSS 24.0 software. All data in tables are expressed as mean values and standard deviations. In figures and tables, different lowercase letters indicate statistically significant differences between groups (*p* < 0.05). All experiments were conducted in triplicate.

## 3. Results and Discussion

### 3.1. Structural Analysis of CGA/COS NPs

Particle size, a crucial parameter for nanoparticles, directly influences physicochemical properties and stability [26]. Table 1 shows the particle size, polydispersity index (PDI), and zeta potential of NPs formed at different concentrations of CGA. The table indicates that at a constant COS content, nanoparticle size first decreased and then increased with rising CGA content. At a CGA content of 2 mg, the nanoparticle size reached its minimum of 90.23 nm, with a PDI value of 0.33. Zeta potential characterizes the surface charge of nanoparticles; a higher absolute value indicates greater stability of the nanoparticle system [27]. The maximum zeta potential of 24.98 mV was observed at a CGA content of 2 mg. The above data indicate that CGA/COS NPs with a CGA content of 2 mg exhibit the smallest particle size and highest stability [28]. This may be attributed to the fact that at this concentration, the negatively charged groups in chlorogenic acid (such as carboxyl and phenolic hydroxyl groups) are fully neutralized by positively charged groups in the chitosan oligosaccharide molecules (such as amino groups). This promotes dense particle contraction. Concurrently, at this addition level, the cross-linking degree between chlorogenic acid and chitosan oligosaccharide molecules is moderate, enhancing nanoparticle stability. A lower chlorogenic acid content results in insufficient charge neutralization and cross-linking, leading to loose particles prone to aggregation. Conversely, excessive amounts cause molecular aggregation and stacking, disrupting the interfacial layer stability and ultimately increasing particle size and destabilizing the system. Moreover, when incorporated into composite materials, nanoparticles with smaller particle sizes disperse more uniformly and more effectively impede dislocation motion within the matrix, significantly enhancing the material’s mechanical properties such as strength and hardness. Consequently, a chlorogenic acid content of 2 mg was selected for the subsequent studies on COS/CGA NPs.

**Figure 1:**
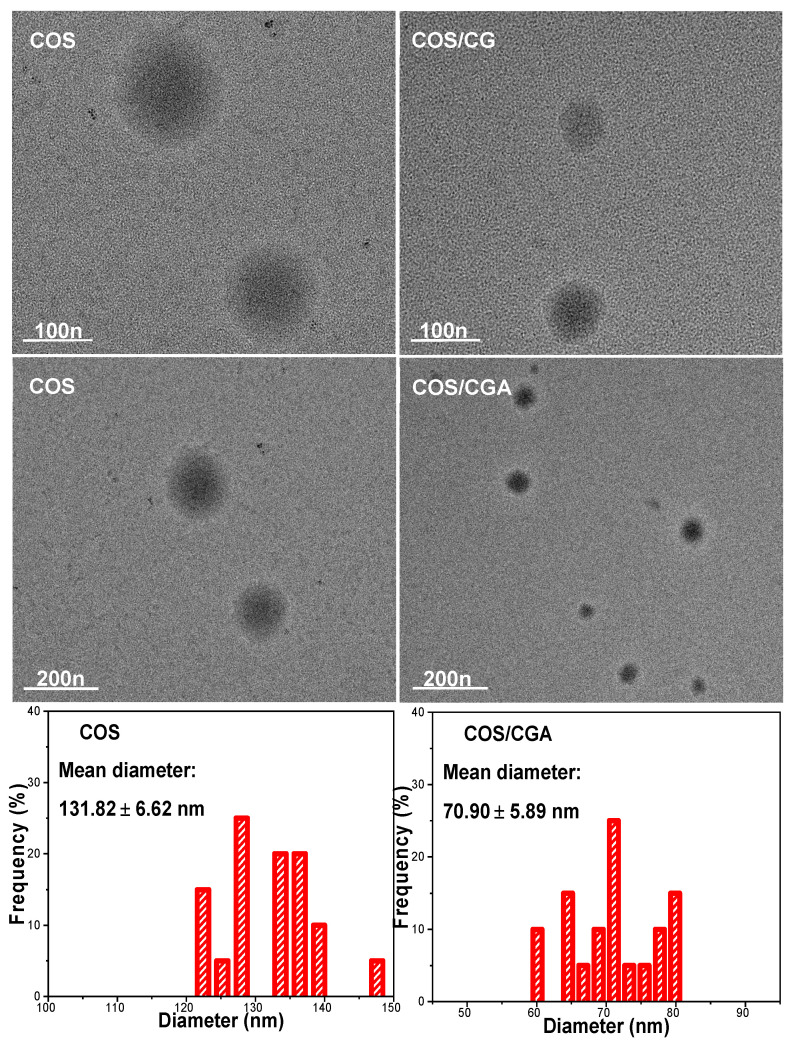
TEM images and diameter distributions of COS NPs and CGA/COS NPs.

Figure 1 presents TEM images of the nanoparticles at different magnifications, revealing their surface morphology. The prepared nanoparticles exhibit spherical shapes with smooth surfaces. The images clearly demonstrate that the nanoparticles maintain good dispersion and relatively uniform particle sizes both before and after CGA incorporation. The measured actual particle size of the COS NPs was 131.82 nm, while that of the COS/CGA NPs was 70.90 nm. The particle size decreases after incorporation, consistent with previous experimental results. This may be attributed to CGA promoting the formation of heterogeneous nanoparticles. CGA can form hydrogen bonds or other weak interactions with amino groups and hydroxyl group on COS molecules, inducing curling or folding of the chains of COS and thereby reducing particle size [29,30]. Notably, it is noteworthy that these particle size results appear smaller compared to data obtained from nanoparticle size and zeta potential analyzer. This discrepancy may arise because electron microscopy was conducted under dry conditions, where nanoparticles were in a contracted state, whereas nanoparticle size and zeta potential analyzer particle size were measured using nanoparticle suspensions, where particles were in a swollen state [31].

### 3.2. Structural Analysis of NPs/CS Composite Films

**Figure 2:**
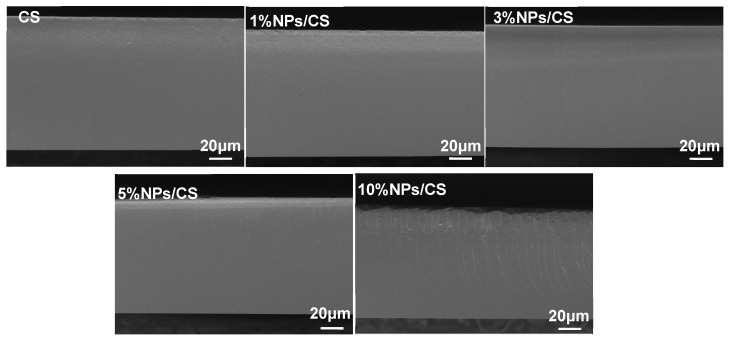
Cross-sectional SEM images of CS film and NPs/CS composite films.

Images of the NPs/CS composite film are shown in Figure S1. All composite films exhibited smooth, uniform, and transparent surfaces, making them suitable for transparent packaging applications. Additionally, SEM analysis of the CS film and NPs/CS composite film revealed their front-surface and cross-sectional morphologies. As shown in the SEM image of the front-surface (Figure S2), the pure chitosan film surface appeared flat with no delamination or cracks observed. The addition of CGA/COS NPs resulted in uniformly dispersed spherical particles on the film surface. As the CGA/COS NPs content increased, the number of spherical particles on the film surface also rose. It should be noted that the large irregular aggregates appearing on the surface of the 10% NPs/CS composite film are likely dust or excess particles, rather than nanoparticles. In addition, it can be observed from the cross-sectional SEM images (Figure 2) that the cross-section of the CS film is uniform and smooth, without wrinkles and voids, and the structure is complete, indicating that chitosan and glycerol are fully integrated. In contrast to the CS film, the composite film incorporating a low concentration of CGA/COS NPs exhibited a smooth surface with reduced thickness. This is because the nanoparticles fill the gaps in the chitosan molecular chain, and there may be hydrogen bonds and other interactions between them and the chitosan molecules, making the molecular arrangement more compact and reducing the looseness of the film structure [32]. However, as nanoparticle content increases, the roughness of the composite film cross-section also rises. When the CGA/COS NPs addition ratio reached 10%, numerous cracks appeared in the composite film cross-section. This may result from enhanced interactions between excessive nanoparticles, leading to agglomeration. Agglomerated nanoparticles form stress concentration points within the film, disrupting its uniform structure [33]. This phenomenon adversely affects the mechanical properties and barrier performance of the composite film.

**Figure 3:**
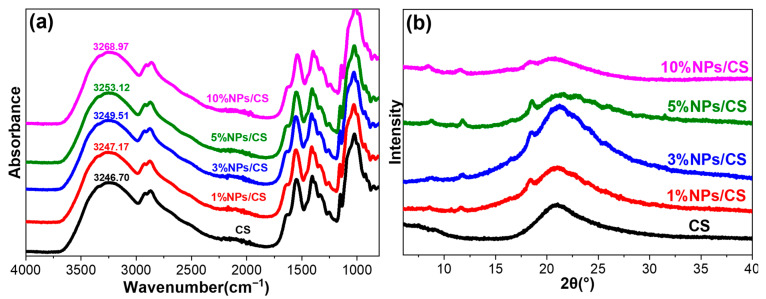
FTIR spectra (**a**) and XRD patterns (**b**) of CS film and NPs/CS composite films.

The FTIR spectra of the CS film and the NPs/CS composite film are shown in Figure 3a. In the spectrum of the CS film, the strong absorption peak at 3246.70 cm^−1^ corresponds to the overlapping stretching vibrations of -OH and -NH groups. The peaks at 1642.07 cm^−1^, 1549.83 cm^−1^, 1406.41 cm^−1^ and 1024.88 cm^−1^ represent the amide group (C=O) stretching vibration, N-H bending vibration, and -C-N and C-O stretching vibrations in the inherent structure of chitosan, respectively [34]. Compared to the FTIR spectrum of the CS film, no new peaks appeared in the composite film with added CGA/COS NPs, indicating no new chemical bonds were formed during composite film preparation [35]. With increasing CGA/COS NPs content, the vibration of -OH groups in the NPs/CS composite film exhibited a significant red shift, shifting from 3247.17 cm^−1^ to 3268.97 cm^−1^, indicating hydrogen bonding between CGA/COS NPs and the chitosan matrix [36]. This crosslinking enhances the cohesive strength of the composite film, leading to improved mechanical properties [37,38].

To investigate the effect of CGA/COS NPs on the crystallinity of the CS composite film, XRD analysis was performed, with results shown in Figure 3b. The CS film exhibits characteristic diffraction peaks near 21°, indicating a typical semi-crystalline state. As can be seen from the figure, the CS film exhibits broader peaks and lower intensity, with the lowest degree of crystallinity. With increasing addition of COS/CGA NPs, the peaks of the NPs/CS composite film narrow and the intensity increases. When the addition reaches 10%, the composite film’s peaks broaden and the intensity decreases. The crystallinity of the NPs/CS composite film first increases and then decreases with increasing CGA/COS NPs content. This phenomenon may arise because low concentrations of NPs act as nucleating agents, providing additional attachment sites for the ordered stacking of chitosan molecular chains. This facilitates the ordered arrangement of molecular chains on their surfaces, thereby enhancing crystallinity [39]. At this stage, enhanced intermolecular forces improve the film’s tensile strength. Concurrently, the dense crystalline structure reduces pathways for gas and water vapor transmission, thereby enhancing barrier properties. However, excessive NPs may agglomerate or become unevenly dispersed, disrupting the ordered arrangement of chitosan molecular chains and consequently damaging the crystalline structure [40]. Consequently, a reduction in crystalline regions and an increase in amorphous regions lead to diminished tensile strength in the film. Furthermore, the loose structure of the amorphous regions weakens barrier properties, a finding consistent with subsequent results concerning OTR, WVP, and mechanical performance.

### 3.3. Performance Analysis of NPs/CS Composite Films

**Figure 4:**
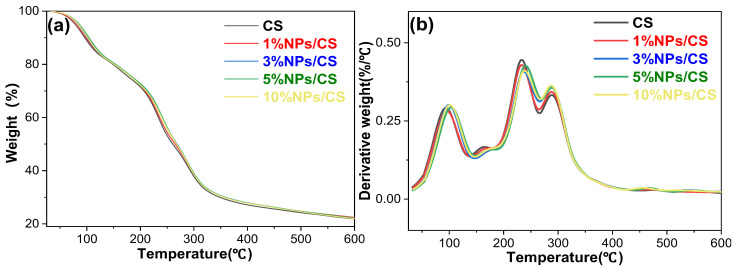
TG (**a**) and DTG (**b**) curves of CS film and NPs/CS composite films.

To evaluate the thermal stability of CS films and NPs/CS composite films, thermogravimetric (TG) analysis (Figure 4a) and differential thermal analysis (DTG) (Figure 4b) were conducted. As shown in Figure 4a, the weight of all films decreased with increasing temperature. The temperature change rate is depicted in Figure 4b. The first stage of thermal degradation occurred between 20 and 100 °C, primarily attributed to the evaporation of moisture within the films [41]. The maximum degradation rates for CS, 1%NPs/CS, 3%NPs/CS, 5%NPs/CS, and 10%NPs/CS films occurred at 92.93 °C, 95.95 °C, 99.99 °C, 103.02 °C, and 102.01 °C, respectively. The incorporation of CGA/COS NPs reduced weight loss in composite films during this stage. The second stage of thermal degradation occurred between 100 and 250 °C, primarily attributed to glycerol decomposition and partial chitosan degradation within the film [42]. At this stage, the temperature corresponding to the maximum degradation rate of the 5%NPs/CS composite film was 241.23 °C, which was slightly higher than the temperature corresponding to the maximum degradation rate of the CS film of 233.16 °C. The third stage occurred at 250–600 °C, and the main stage was the complete decomposition of chitosan [43]. In summary, the incorporation of COS/CGA NPs resulted in a slight improvement in the thermal stability of the CS composite film, with the 5%NPs/CS composite film exhibiting the highest thermal stability. This may result from an appropriate amount of nanoparticles improving the film’s thermal stability through intermolecular hydrogen bonding [44]. In this study, although the degradation temperature is significantly higher than the conventional operating range for refrigerated food packaging (0–4 °C), the enhancement in thermal stability holds considerable practical significance. On the one hand, the industrial production of food packaging films involves processes such as extrusion and heat sealing, typically requiring resistance to temperatures of 150–200 °C. Improved thermal stability prevents thermal degradation during film processing, thereby reducing the generation of harmful substances. On the other hand, films may encounter environmental temperature fluctuations such as summer heat during prolonged storage. Enhanced thermal stability reduces their aging rate, safeguards performance consistency during use, and extends their shelf life.

Water content, swelling degree, and water solubility are key indicators affecting film water resistance. As shown in Table 2, the CS film exhibited the highest water content (34.69 ± 0.41%) and swelling degree (68.30 ± 1.51%) due to its abundant hydrophilic groups. After adding CGA/COS NPs, the water content and swelling degree of the film decreased. This is attributed to the formation of hydrogen bonds between nanoparticles and chitosan molecules, which reduces the groups in chitosan capable of binding water molecules [45]. Simultaneously, nanoparticles fill the interstitial spaces between chitosan molecules, promoting the compact arrangement of chitosan molecular chains. This reduces the film’s porosity and pore size, diminishing water permeation pathways and retention space, thereby lowering the film’s water content and swelling degree [46]. This finding is consistent with the SEM results. However, when the NPs content reached 10%, the film’s moisture content, swelling degree, and water solubility actually increased. This may be attributed to the interaction between excess NPs, which readily leads to agglomeration, coupled with the introduction of excessive hydrophilic groups. Both factors contribute to enhanced hydrophilicity and reduced water resistance in the film.

**Figure 5:**
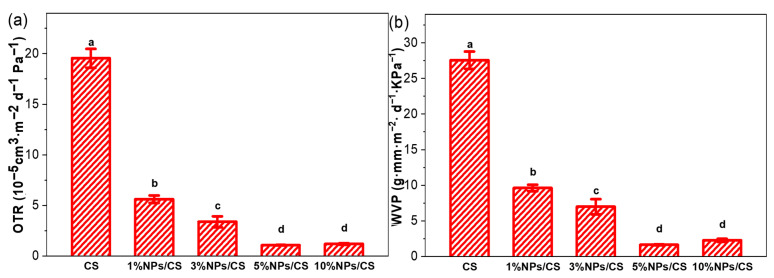
Oxygen transmission rate (OTR) (**a**) and Water vapor permeance (WVP) (**b**) of CS film and NPs/CS composite films. Different superscript letters indicate significant differences at *p* ≤ 0.05 (*n* = 3).

OTR and WVP values are two critical parameters for food packaging materials, typically influenced by environmental conditions, film structure, and biopolymer properties. Low levels of OTR and WVP values can impede the exchange of water vapor and oxygen between packaged food and its surroundings, thereby slowing the oxidative deterioration of fresh food [47]. As shown in Figure 5, the CS film exhibited an OTR value of (19.54 ± 0.93) × 10^−5^ cm^3^/(m^2^·d·Pa) and a WVP value of (27.55 ± 1.21) g·mm/(m^2^·kPa), owing to the abundant hydrophilic groups in the chitosan structure that exhibit strong affinity for water molecules. After adding CGA/COS NPs, both OTR and WVP values of the composite film decreased significantly. Among them, the 5% NPs/CS composite film exhibited the lowest OTR and WVP values, at (1.06 ± 0.03) × 10^−5^ cm^3^/(m^2^·d·Pa) and (1.65 ± 0.08) g·mm/(m^2^·d·kPa), respectively. This reduction is attributed to hydrogen bonding interactions between the nanoparticles and chitosan, which decrease the number of hydrophilic groups in the system, thereby lowering the WVP value [45]. The presence of nanoparticles forces diffusing gases or liquids in the environment to follow tortuous paths through the composite film, increasing the path length [48]. Simultaneously, the addition of CGA/COS NPs renders the composite film structure more compact and dense, effectively blocking water vapor and oxygen transmission, consistent with FTIR, XRD and SEM results.

**Figure 6:**
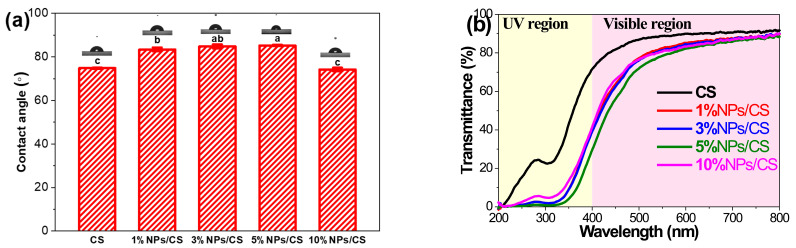
Water contact angles (**a**) and UV−Vis transmittances (**b**) of CS film and NPs/CS composite films. Different superscript letters indicate significant differences at *p* ≤ 0.05 (*n* = 3).

Surface wettability of the CS film and the NPs/CS composite film was evaluated by measuring the static contact angle, with results shown in Figure 6a. The figure shows that the static contact angle of the CS film is 74.8°. After adding CGA/COS NPs, the static contact angle of the composite film increases. This is because the nanoparticles fill the gaps between chitosan molecules, not only making water molecule diffusion inward more difficult but also making the composite film structure more compact and dense, thereby enhancing the film’s hydrophobicity [49]. At a 5% loading, the composite film exhibited the maximum static contact angle of 85.2° and the highest hydrophobicity. However, when the NPs loading reached 10%, the static contact angle decreased. This decline may result from excessive interactions between NPs, leading to agglomeration, or the introduction of too many hydrophilic groups, which increases the film’s hydrophilicity. Films with high water resistance are advantageous for use as food packaging materials [50].

Ultraviolet radiation accelerates food oxidation. Excellent UV resistance effectively prevents photo-oxidation, preserving food texture and flavor [50]. As shown in Figure 6b, all NPs/CS composite films exhibit strong UV resistance. Data in Table 3 indicates that the 5%NPs/CS composite film has a transmittance of 0.79% at 300 nm, approaching zero and significantly lower than the 22.69% of the CS film, demonstrating the strongest UV resistance. This likely stems from the absorption of ultraviolet light by multiple conjugated double bonds and hydroxyl groups within the chlorogenic acid molecular structure. Additionally, since the size of nanoparticles is similar to the wavelength of ultraviolet light, scattering occurs when ultraviolet light is incident, thereby reducing the intensity of ultraviolet light directly passing through the film and exhibiting excellent ultraviolet shielding effects [51]. The opacity values of the films are presented in Table 3, where higher values indicate lower transparency. The opacity of transparent food packaging typically falls below 5 [52]. The CS film exhibited an opacity of 1.21. Following the incorporation of COS/CGA NPs, transparency decreased slightly, with opacity values increasing to 1.36, 1.48, 1.73, and 1.72, respectively. This may be attributed to the accumulation of NPs within the polymer matrix, leading to light scattering and reduced light transmittance. The opacity value of the 5% NPs/CS composite film (1.73) remains substantially below the 5 required for transparent food packaging, potentially positively influencing consumer decision-making regarding the product [53]. Overall, the 5%NPs/CS composite film demonstrated the strongest barrier properties against moisture and UV radiation, showing great potential for food packaging applications.

Table 3 indicates that the film thickness ranges from 0.090 to 0.123 μm. Compared to the CS film, the thickness of the NPs/CS composite film decreased, likely due to reduced moisture content following the addition of CGA/COS NPs. Uniaxial tensile testing was conducted to evaluate the mechanical properties of the films. As shown in Table 3, the CS film exhibited a tensile strength of 13.57 MPa and an elongation at break of 18.08%. The mechanical strength of the NPs/CS composite films significantly surpassed that of the CS film. Specifically, the tensile strength and elongation at break of the 5% NPs/CS composite film were 34.64 MPa and 90.06%, respectively, representing a significant improvement over the CS film. This enhancement stems partly from the physical crosslinking formed by nanoparticles tightly binding to chitosan chains via hydrogen bonds and van der Waals forces, enabling efficient stress transfer from the matrix to the nanoparticles. Additionally, the large specific surface area of nanoparticles strengthens interfacial interactions, thereby significantly increasing the elongation at break and enhancing the mechanical properties. [54]. When the nanoparticle content reached 10%, the mechanical properties of the composite film exhibited a certain degree of decline compared to the 5%NPs/CS composite film. This was attributed to partial aggregation of CGA/COS NPs, which altered the microstructure of the film. Additionally, uneven stress transfer between the particles and the chitosan matrix led to the formation of agglomerates within the film matrix. These agglomerates restricted the movement of polymer chains, thereby reducing the mechanical strength of the film [38], consistent with the XRD results. The elongation at break serves as an indicator of a film’s tensile properties, reflecting its capacity for deformation under applied stress. The 5% NPs/CS composite film exhibits a higher elongation at break, thereby demonstrating superior ductility. Concurrently, the tensile strength of the 5% NPs/CS film increases in tandem with its elongation at break relative to the CS film, indicating it possesses both rigidity and toughness with reduced brittleness. This renders it suitable for use as packaging material.

**Figure 7:**
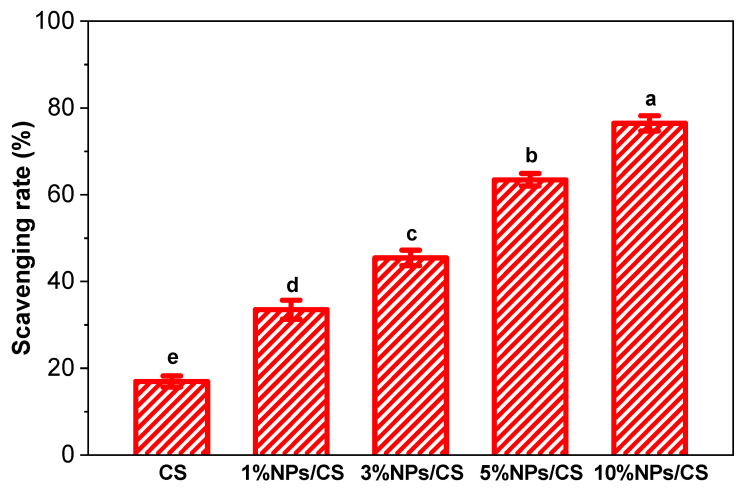
Antioxidant activity of CS film and NPs/CS composite films. Different superscript letters indicate significant differences at *p* ≤ 0.05 (*n* = 3).

Oxidation is a major factor causing flavor deterioration and spoilage in food, making high antioxidant properties crucial for food packaging coatings [50]. The antioxidant activity of CS films and NPs/CS composite films was evaluated using the DPPH radical scavenging assay. As shown in Figure 7, the CS film exhibited a DPPH radical scavenging rate of 16.95%, primarily influenced by the -NH_2_ and -OH groups in chitosan, indicating relatively weak antioxidant activity [55]. After incorporating CGA/COS NPs, the DPPH radical scavenging activity of the composite film significantly increased from 33.52% to 76.47%. This enhancement stems from chlorogenic acid, a polyphenolic compound that acts as a potent antioxidant. Its molecular structure contains abundant phenolic hydroxyl groups capable of donating hydrogen atoms to scavenge radicals [56]. The antioxidant activity of the film showed a positive correlation with the added nanoparticle concentration, demonstrating dose-dependent antioxidant capacity. The DPPH assay employed in this study utilized a system where the film was directly immersed in an ethanol solution. Whilst this method does not fully replicate the actual storage conditions of fresh *Pleurotus geesteranus*, ethanol, as a polar solvent, can to some extent simulate the water and polar components within the *Pleurotus geesteranus* tissue. Consequently, the experimental results possess reference value. The results demonstrate that the NPs/CS composite film can serve as an active packaging material to effectively limit or reduce oxidative damage to food, thereby extending its shelf life.

### 3.4. Application in Pleurotus geesteranus Preservation

The 5%NPs/CS composite film exhibited the highest thermal stability, water resistance, mechanical properties, and barrier performance against oxygen and ultraviolet light, while also demonstrating good antioxidant activity. Consequently, the 5%NPs/CS composite film was selected for subsequent *Pleurotus geesteranus* preservation experiments.

**Figure 8:**
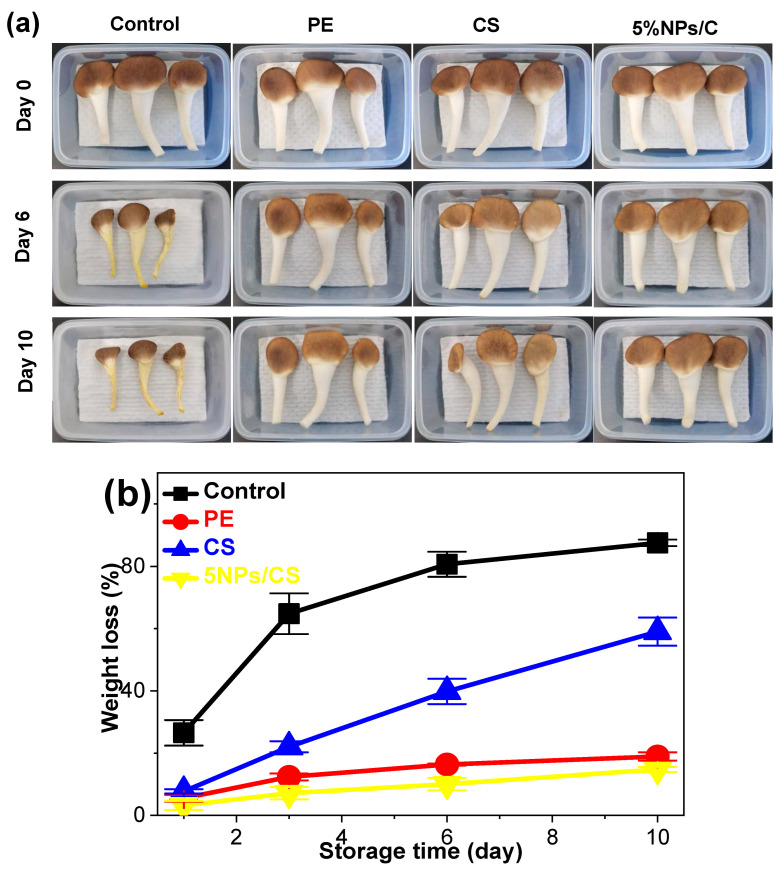
Changes in visual appearance (**a**) and mass loss rate (**b**) of *Pleurotus geesteranus* packaged in different films.

As shown in Figure 8a, after 6 days of storage, the control group’s *Pleurotus geesteranus* exhibited severe shriveling of the fruiting body, yellowing of the stipes, and numerous cracks on the pileus. Although PE and CS films provided some preservation effects, by day 10, treated mushrooms developed whitening of the pileus, softened texture, shriveled fruiting bodies, and off-odors. In contrast, *Pleurotus geesteranus* treated with the 5%NPs/CS composite film demonstrated optimal preservation in color, morphology, and odor. Pileus color showed no significant change, no off-odors were detected, and the mushroom bodies remained plump and elastic, with minimal shriveling, indicating superior quality. The preservation efficacy of the 5%NPs/CS composite film surpassed that of commercially available PE and CS films. The weight loss rates of the *Pleurotus geesteranus* further corroborate these findings. As shown in Figure 8b, weight loss rates gradually increased across all groups with extended storage duration. This resulted from the mushrooms’ respiration and water evaporation during storage, exacerbated by their thin epidermal structure, which cannot prevent high transpiration rates [57]. The control group exhibited a sharp increase in weight loss, reaching 87.53% throughout storage. In contrast, weight loss rates were significantly mitigated for oyster mushrooms packaged with PE film, CS film, and the 5%NPs/CS composite film. Among these, mushrooms treated with the 5%NPs/CS composite film maintained the highest moisture content, showing a weight loss rate of only 14.76% by day 10. This is attributed to its high tensile strength, high water resistance, and low water vapor permeability. The high tensile strength prevents the composite film from tearing during use and provides effective isolation from external environments. The high water resistance ensures the composite film structure remains intact despite moisture exposure, safeguarding its performance from significant degradation caused by moisture generated during the mushroom’s normal physiological metabolism. The low water vapor permeability prevents excessive moisture loss from the mushroom surface, thereby preserving freshness [58]. Moreover, the potent antioxidant activity of CGA scavenges free radicals generated during *Pleurotus geesteranus* respiration, thereby delaying lipid peroxidation in cell membranes and maintaining cellular structural integrity to minimize moisture loss. Concurrently, as documented in existing literature, CGA exhibits antimicrobial properties [11], inhibiting the growth and reproduction of spoilage microorganisms. This reduces damage to *Pleurotus geesteranus* tissue caused by microbial metabolism, further diminishing weight loss and thus playing a positive role in preserving *Pleurotus geesteranus* freshness. In summary, the 5%NPs/CS composite film demonstrates significant application potential in the food packaging industry.

## 4. Discussion

The chitosan-based edible films loaded with CGA/COS nanoparticles prepared in this study possess inherent advantages in food safety and comply with relevant food contact material regulations. Firstly, chlorogenic acid is a natural plant extract. As a functional component in health foods, its safety assessment is regulated and guaranteed by the national standard GB/T 22250-2025 [59]: Determination of Chlorogenic Acid in Health Foods. Chitosan oligosaccharides, a degradation product of chitin, are recognized as a safe food ingredient with excellent biocompatibility and degradability. Furthermore, the film preparation process employs no toxic or harmful additives, adheres to green and environmentally friendly production standards, and meets the fundamental requirements of China’s GB 4806 series standards, thereby establishing a regulatory foundation for commercial application.

## 5. Conclusions

This study successfully prepared chitosan composite film loaded with chitosan oligosaccharide–chlorogenic acid nanoparticles. It investigated the effects of CGA/COS NPs as additives on the structure and comprehensive properties of chitosan-based films, as well as the preservation efficacy of NPs/CS composite films for *Pleurotus geesteranus*. Results indicate that CGA/COS NPs exhibit spherical morphology, with minimal particle size and optimal stability achieved at a chlorogenic acid content of 2 mg. The NPs significantly influence composite film properties. SEM, FTIR, and XRD analyses demonstrate excellent compatibility between NPs and the film matrix, with NPs forming hydrogen bonds with chitosan to enhance cohesive strength and improve composite film performance. The 5%NPs/CS composite film exhibited optimal comprehensive properties. Adding 5%NPs improved the film’s thermal stability, water resistance, and barrier properties against oxygen and ultraviolet radiation. It also enhanced the mechanical properties of the composite film, increasing tensile strength from 13.57 MPa to 34.64 MPa and elongation at break from 18.08% to 90.06%. Furthermore, CGA/COS NPs significantly enhanced the film’s antioxidant activity, with DPPH radical scavenging activity increasing from 16.95% to 76.47%, exhibiting a positive dose-dependent correlation. Preservation experiments on *Pleurotus geesteranus* revealed that the 5%NPs/CS composite film exhibited optimal preservation performance, effectively delaying weight loss and extending shelf life. This study successfully developed an environmentally friendly, high-performance edible composite film, offering a valuable new approach to addressing preservation challenges for perishable agricultural products like *Pleurotus geesteranus* and advancing innovation in the green food packaging industry.

## Figures and Tables

**Table 1:** Effect of CGA concentration on particle size, zeta potential, and encapsulation efficiency of the NPs. Different superscript letters indicate significant differences at *p* ≤ 0.05 (*n* = 3).

CGA (mg)	Particle Size (nm)	Polydispersity Index	Zeta Potential (mV)
0.00	165.59 ± 11.70 ^a^	0.39 ± 0.03 ^a^	18.24 ± 0.73 ^d^
1.00	131.35 ± 7.15 ^b^	0.37 ± 0.02 ^ab^	22.11 ± 0.55 ^c^
2.00	90.23 ± 4.32 ^d^	0.33 ± 0.01 ^c^	24.98 ± 0.66 ^a^
3.00	113.10 ± 5.50 ^c^	0.39 ± 0.02 ^a^	23.56 ± 0.22 ^b^
4.00	128.02 ± 7.34 ^b^	0.35 ± 0.01 ^bc^	24.19 ± 0.90 ^ab^

**Table 2:** Moisture content (a), Swelling degree (b) and Water solubility (c) of CS film and NPs/CS composite films. Different superscript letters indicate significant differences at *p* ≤ 0.05 (*n* = 3).

Sample	Moisture Content	Swelling Degree	Water Solubility
CS	34.69 ± 0.41 ^a^	68.30 ± 1.51 ^a^	22.34 ± 1.43 ^ab^
1%NPs/CS	32.76 ± 0.94 ^b^	66.66 ± 0.43 ^ab^	20.40 ± 0.67 ^c^
3%NPs/CS	32.47 ± 0.48 ^b^	63.37 ± 0.99 ^c^	21.40 ± 0.16 ^bc^
5%NPs/CS	31.07 ± 0.09 ^c^	56.29 ± 1.59 ^d^	23.52 ± 0.19 ^a^
10%NPs/CS	33.34 ± 0.73 ^b^	64.69 ± 1.19 ^bc^	23.29 ± 0.69 ^a^

**Table 3:** Thickness, Mechanical properties, Optical properties and Opacity values of CS film and NPs/CS composite films. Different superscript letters indicate significant differences at *p* ≤ 0.05 (*n* = 3).

Sample	Thickness (mm)	Mechanical Properties		Optical Properties (%)		Opacity Values
		Tensile Strength (MPa)	Elongation at Break (%)	300 nm	450 nm	
CS	0.123 ± 0.006 ^a^	13.57 ± 0.68 ^e^	18.08 ± 6.14 ^d^	22.69	81.75	1.21 ± 0.06 ^d^
1%NPs/CS	0.117 ± 0.006 ^ab^	16.95 ± 1.63 ^d^	28.74 ± 7.62 ^d^	1.99	63.79	1.36 ± 0.03 ^c^
3%NPs/CS	0.103 ± 0.006 ^bc^	26.65 ± 0.57 ^c^	44.25 ± 2.10 ^c^	1.99	62.50	1.48 ± 0.04 ^b^
5%NPs/CS	0.090 ± 0.010 ^c^	34.64 ± 1.27 ^a^	90.06 ± 8.48 ^a^	0.79	56.06	1.73 ± 0.08 ^a^
10%NPs/CS	0.093 ± 0.012 ^c^	29.68 ± 0.87 ^b^	58.68 ± 3.55 ^b^	4.80	66.16	1.72 ± 0.06 ^a^

## Data Availability

The original contributions presented in this study are included in the article/supplementary material. Further inquiries can be directed to the corresponding authors.

## Supplementary Materials

The following supporting information can be downloaded at: https://www.mdpi.com/article/10.3390/foods15020221/s1, Figure S1: Surface morphology of CS film and CS-NPs composite films; Figure S2: Front-surface SEM images of CS film and NPs/CS composite films.

## Abbreviations

The following abbreviations are used in this manuscript:

CGA/COS NPs	Chlorogenic acid-chitosan oligosaccharide nanoparticles
NPs/CS	Nanoparticles/chitosan
