# Supplementary material for: Preparation of Chitosan Composite Film Loaded with Chlorogenic Acid–Chitosan Oligosaccharide Nanoparticles and Its Application in Preservation of Pleurotus geesteranus
Source: Foods. 2026 Jan 8;15(2):221. doi: 10.3390/foods15020221 (PMC12840070; doi:10.3390/foods15020221)
Supplement: Supplementary file 1 [file foods-15-00221-s001.zip › foods-4050364-supplementary.pdf]

Supplementary Material

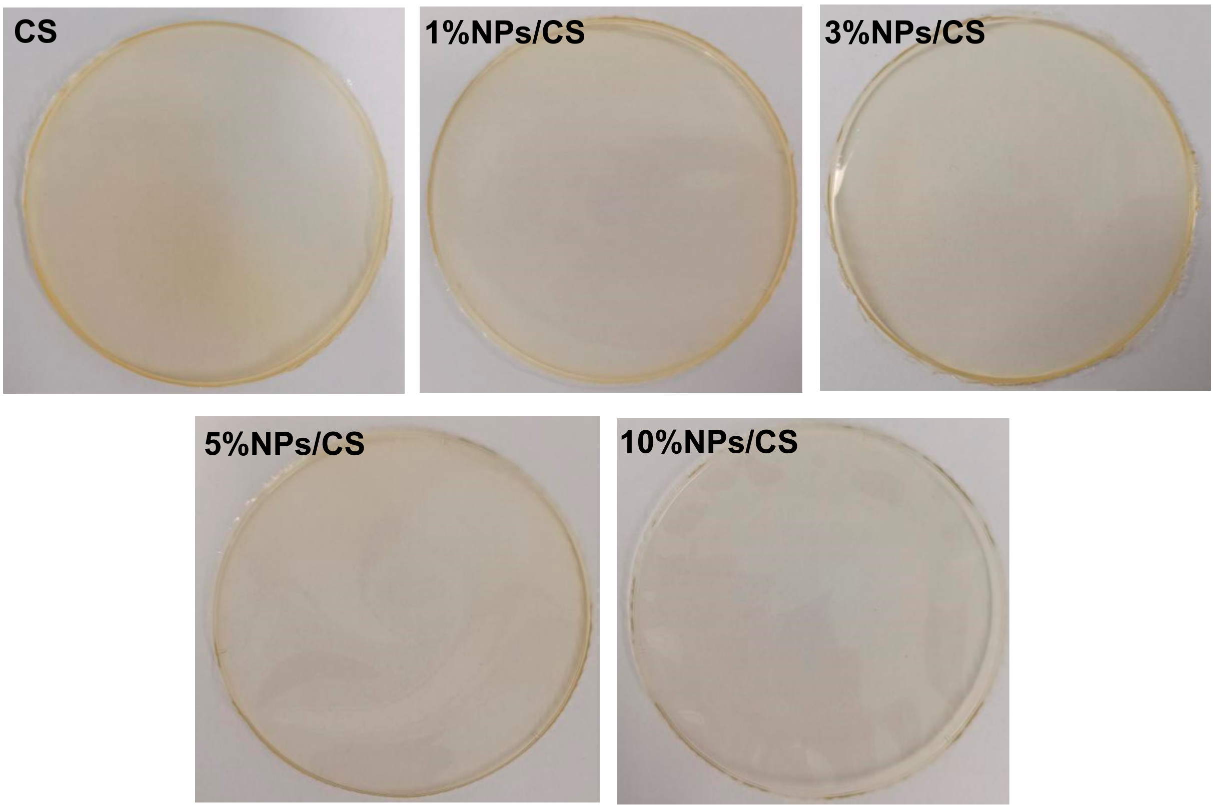

Figure.S1 Surface morphology of CS film and CS-NPs composite films.

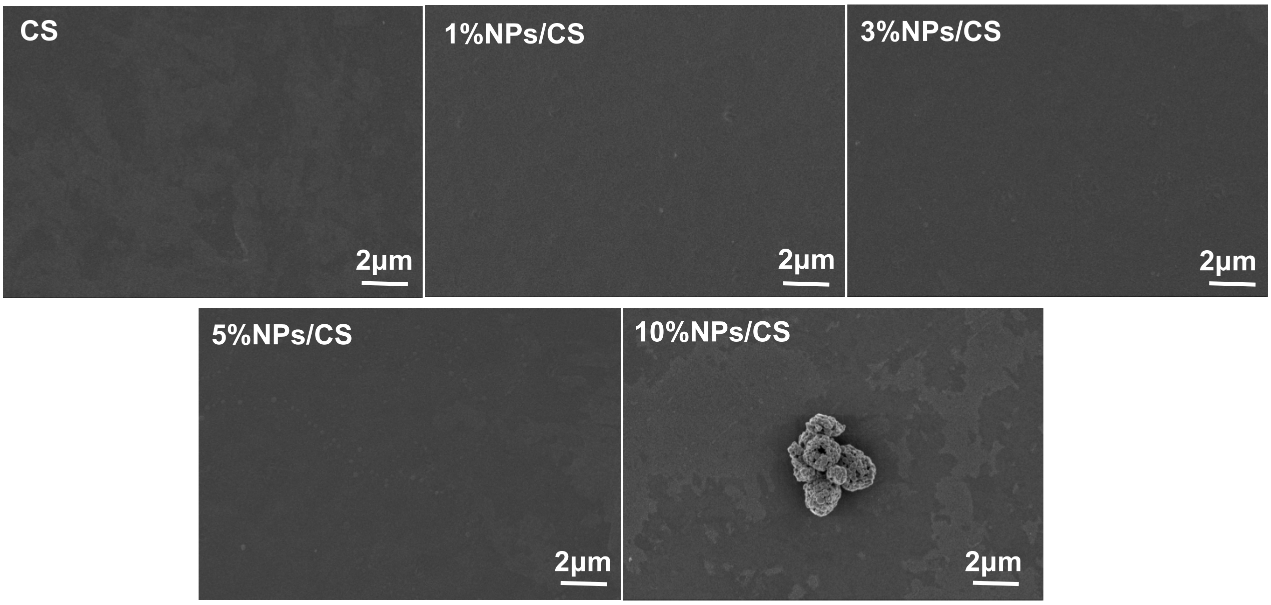

Figure.S2 Front-surface SEM images of CS film and NPs/CS composite films.
